# Serum and salivary adiponectin levels as predictive markers for diabetes mellitus in children with a family history of diabetes

**DOI:** 10.25122/jml-2023-0023

**Published:** 2023-10

**Authors:** Maryam Hamid Merzah, Ameena Ryhan Diajil

**Affiliations:** 1 Department of Oral Diagnosis, Oral Medicine, College of Dentistry, University of Baghdad, Baghdad, Iraq

**Keywords:** saliva, serum, adiponectin, family history, children, body mass index, ADP: Adiponectin, BMI: Body Mass Index, FHD: Family History of Diabetes, DM: Diabetes Mellitus

## Abstract

Diabetes mellitus (DM) is a chronic, metabolic condition marked by defects in insulin production, action, or both. Environmental and genetic factors can contribute to the onset of diabetes mellitus. Adiponectin, a hormone affecting pancreatic beta cell proliferation, has emerged as a potential indicator of diabetes risk. This cross-sectional study aimed to evaluate serum and salivary adiponectin levels as predictors of diabetes mellitus in children with/without a family history of diabetes mellitus. The study was conducted at Al-Zahra Hospital in Najaf city and included 125 children aged 5 to 16. Data on demographics, including name, age, and gender, were collected, and body mass index (BMI) was assessed. Serum and salivary adiponectin levels were measured and analyzed in relation to family history and BMI. Children with a family history of DM had high serum adiponectin (ADP) levels. Serum adiponectin levels were significantly higher in children with first-degree relatives having a history of diabetes mellitus, except for cases involving mothers and other relatives with diabetes mellitus history (p<0.05). Furthermore, serum adiponectin levels were higher in obese children. Salivary adiponectin levels were significantly elevated in children with a maternal family history of diabetes (p=0.01), while no significant correlation was found with BMI. A significant negative correlation (r=-0.180, p=0.05) between salivary and serum adiponectin concentrations was observed. Compared to children with a normal, healthy weight, children with obesity had decreased salivary adiponectin levels and increased serum adiponectin levels.

## INTRODUCTION

Chronic hyperglycemia, a complex metabolic disorder known as diabetes mellitus (DM), is characterized by deficiencies in insulin secretion, action, or both [1]. Insufficient insulin action on target tissues leads to anomalies in protein, lipid, and carbohydrate metabolism, resulting in compromised hormonal pathways, contributing to the complexity of the disease [2, 3]. Obesity and diabetes mellitus result in significant health challenges worldwide [4]. Maintaining a balanced diet and nutrition is essential in preventing diabetes and related non-communicable diseases [5].

Saliva has dynamic diagnostic properties that enable the detection of oral and systemic diseases by analyzing salivary biomarkers [6]. Type 1 diabetes (T1DM), which can manifest at any age and accounts for 10 to 15% of all diabetes cases, is characterized by autoimmune processes involving islet-specific auto-reactive CD4+ and CD8+ T lymphocytes [7-10]. Children of mothers with type 1 diabetes have a 1-4% chance of developing the condition, whereas children of fathers with type 1 diabetes have a 10% chance. Despite this genetic predisposition, 80-85% of new cases involve people with no known family history of diabetes [11]. Nevertheless, a family history of type 1 diabetes is associated with an increased risk of developing type 2 diabetes, often with a later onset [12]. Environmental elements are crucial in the pathophysiology of T1DM [13].

In contrast, type 2 diabetes (T2DM), often referred to as non-insulin-dependent or adult-onset diabetes, is characterized by insulin resistance and a relative insulin deficiency [14]. The development of type 2 diabetes is influenced by a complex interplay of genetic, environmental, and behavioral risk factors [15]. A family history of diabetes is a significant predictor of T2DM, with individuals having relatives with diabetes being 2-4 times more likely to develop it [16]. The prevalence of overweight individuals among those with diabetes was slightly higher than among non-diabetics, while the incidence of underweight individuals was lower [17].

Adipocytes within white adipose tissue secrete a protein known as adiponectin, which regulates several physiological functions, including insulin sensitivity, appetite control, energy balance, and inflammatory responses [18, 19]. Reduced plasma or serum adiponectin levels have been linked to the development of type 2 DM and insulin resistance [20, 21]. The adiponectin gene (ADIPOQ), which encodes adiponectin, is located on chromosome 3 q27.3 [22]. In salivary adipocytes, research has revealed a higher presence of adipocytes in saliva produced by fat cells, particularly in the parotid acinar and interstitial tissue of T2DM patients, which is rich in lipids [23]. Salivary adiponectin levels in obese children were markedly lower compared to those of individuals with normal healthy weight [24]. This study aimed to evaluate serum and salivary adiponectin levels as predictors of diabetes mellitus in children with/without a family history of diabetes mellitus.

## MATERIAL AND METHODS

### Study design

This cross-sectional study included 125 children ranging in age from 5 to 16 and was conducted at Al-Zahraa Hospital in Najaf city. Inclusion criteria comprised children aged 5 to 16, with or without a family history of diabetes mellitus, and those with informed consent from their parents or guardians. Exclusion criteria encompassed children with difficulty opening mouth, recent antibiotic use within the past month, systemic illnesses, orthodontic appliances, and intellectual disabilities.

### Sample collection

#### Blood sample collection

Venous blood samples were collected, with each sample tube meticulously labeled with the subject's name. After clotting at 37°C for 15-20 minutes, the samples underwent centrifugation at 3,000 rpm for 15 minutes to isolate serum. The serum was divided into portions and transferred to sterile microcentrifuge tubes. These tubes were transported to AL-Hakeem Hospital and stored in a deep freezer at -80°C.

#### Saliva sample collection

Unstimulated whole saliva was obtained from the children using the spitting method, with collections conducted between 9 AM and 11 AM. The saliva samples were transported to AL-Hakeem Hospital and stored in a deep freezer at -80°C until further analysis. The collected saliva was centrifugated at 3,000 rpm for 10 minutes, separating the clear supernatant, which was subsequently stored in a deep freezer at -80°C until analysis.

### Analysis method

The sandwich-enzyme linked immune-sorbent assay (ELISA) test for adiponectin was conducted using reagents following the manufacturer's instructions (Elabscience^®^). In brief, samples (or standards) and biotinylated detection antibodies specific for human ADP/Acrp30 were added to the micro-ELISA plate wells. This allowed for the binding of human ADP/Acrp30 to the specific antibody. Avidin-horseradish peroxidase (HRP) conjugate was added to each microplate and washed. The enzyme-substrate reaction was halted by adding a stop solution, resulting in a color change to yellow. The optical density (OD) was measured by a spectrophotometer at 450±2 nm wavelength. The OD value was proportional to the concentration of Human ADP/Acrp30. To determine the ADP/Acrp30 concentration in the samples, the OD of the samples was compared to a standard curve for calibration.

### Statistical Analysis

Data were analyzed using SPSS software version 25. Descriptive statistics, including averages, frequencies, and percentages, were used for categorical data. Numerical data were described using mean and standard deviation. The normal distribution of continuous data was assessed using the Shapiro-Wilk test. The Chi-square test was employed for comparing frequencies, while bivariate Pearson's correlation test was used to evaluate correlations between variables. The correlation coefficient (r) measured the strength and direction of correlations. The F-test was utilized for comparisons involving more than two groups, and t-tests were used to compare means between two independent groups. Statistical significance was defined as a p-value of 0.05 or lower.

## RESULTS

There was no significant association between serum ADP and family history or BMI except for children with a first-degree relative history of diabetes (fathers, sisters, and brothers). These children had higher ADP serum levels than those without a family history (p=0.04, p=0.03, p=0.03) (Table 1).

**Table 1:** Relationship between study variables and serum ADP

Variables			Mean ADP ng/ml	±SD	T-Test or F Test	p-value
History of DM first-degree	Father	Yes	11.33	1.67	4.26	0.04
		No	10.02	2.05		
	Mother	Yes	10.65	1.40	0.53	0.46
		No	10.10	2.09		
	Sister	Yes	10.17	-	4.44	0.03
		No	5.89	2.02		
	Brother	Yes	10.17	.	4.44	0.03
		No	5.89	2.02		
Second degree	Uncle	Yes	10.59	1.84	2.12	0.14
		No	9.98	2.10		
	Grandmother	Yes	10.21	2.10	0.20	0.65
		No	10.05	2.01		
	Grandfather	Yes	10.41	2.01	1.71	0.19
		No	9.93	2.06		
BMI	Underweight		9.73	1.81	1.51	0.21
	Normal		10.19	2.42		
	Overweight		8.91	2.59		
	Obese		10.49	1.93		

Regarding family history and BMI, there was no significant difference in the level of salivary ADP in children, except for those with mothers having a history of DM, who had higher salivary ADP levels (p=0.01) (Table 2).

**Table 2:** Relationship between salivary ADP and study variables

Variables			Mean ADP ng/ml	±SD	T-Test or F Test	p-value
History of DM first-degree relative	Father	Yes	6.21	4.20	1.17	0.28
		No	7.76	4.55		
	Mother	Yes	7.87	4.02	5.81	0.01
		No	3.96	4.47		
	Sister	Yes	3.05		1.02	0.31
		No	7.66	4.53		
	Brother	Yes	3.05		1.02	0.31
		No	7.66	4.53		
Second-degree relative	Uncle	Yes	7.75	5.23	0.03	0.85
		No	7.58	4.30		
	Grandmother	Yes	7.16	4.71	1.38	0.24
		No	8.12	4.30		
	Grandfather	Yes	7.78	4.71	0.11	0.73
		No	7.50	4.42		
BMI	Underweight		8.59	4.14	0.88	0.44
	Normal		7.22	4.73		
	Overweight		7.73	3.18		
	Obese		7.12	4.76		

There was a negative significant correlation between ADP levels in saliva and serum (r=-0.180, p=0.05). However, there was no significant correlation between BMI and ADP levels in serum or saliva (Table 3).

**Table 3:** Pearson correlation coefficient (r) for adiponectin marker

Parameters	ADP Serum	BMI
ADP Saliva	-0.180*	0.070
p-value	0.05	0.451
ADP Serum		-0.120
p-value		0.191

* Correlation is significant at a 0.05 level

## DISCUSSION

In this study, children with a first-degree relative with a history of diabetes mellitus (DM) generally had significantly higher serum adiponectin levels. However, this trend was not observed in children whose mothers or other relatives had a history of DM. This disagrees with Joung *et al*. [25], who reported that adiponectin concentrations were independently related to a family history of diabetes. This may be explained by the fact that children of diabetic parents often exhibit signs of excess body lipid as early as childhood, and the development of insulin resistance syndrome in childhood can accelerate into young adulthood [26]. Serum adiponectin was higher in children with obesity. However, this contradicts another study by Abdella *et al*. [27], which found that serum adiponectin levels decreased significantly with increasing obesity due to visceral adiposity and insulin resistance [28].

Our study observed that salivary adiponectin was higher and significantly associated with children with a family history of DM, specifically with mothers. However, there was no significant association with other first and second-degree relatives. Adipose tissue and other tissues, including the salivary gland and mammary epithelial cells, produce most adiponectin [29]. Regarding salivary adipokines, it was discovered that the presence of more adipokines in saliva produced by fat cells was caused by the parotid acinar and interstitial tissue of T2DM patients, which is rich in lipids [23]. To our knowledge, no previous study has explored salivary adiponectin in children from families with a history of DM. Compared to children with normal healthy weight, salivary adiponectin levels decrease in obese children [30]. This suggests that salivary adiponectin could be a diagnostic indicator of obesity [31].

Our study also found a negative and significant correlation between serum adiponectin and salivary adiponectin levels. This outcome is consistent with a study by Desai *et al*. [32], which observed a significant association between salivary and serum adiponectin levels. Adiponectin is an adipocyte-derived protein with a high plasma concentration [33]. Salivary adipokines were shown to be more abundant in the parotid acinar and interstitial tissue of T2DM patients, which are richer in lipids and responsible for the higher levels of adipokines secreted by fat cells [23]. According to this study, there was no significant correlation between BMI and ADP saliva, which agrees with another study [34] that outlined no significant correlation between adiponectin levels in saliva and BMI. Also, there was no significant correlation between BMI and ADP serum. This disagrees with other studies [35-37] that observed a significant negative correlation between plasma adiponectin levels and BMI. Additionally, our findings disagree with Lina *et al*. [38], who noted a non-significant correlation between adiponectin and BMI.

## CONCLUSION

Salivary adiponectin levels in obese children were lower compared to children with an average, healthy weight. In contrast, serum adiponectin levels were higher in obese children. Children with a positive family history of diabetes may exhibit alterations in salivary and serum parameters, particularly in adiponectin. These changes could potentially serve as indicators of certain hormonal and metabolic changes that contribute to the development of hereditary diseases.
